# “The Strange Case of Dr Pump and Mr Acardiac”: The Twin Reversed Arterial Perfusion (TRAP) Sequence in Two Monochorionic Monoamniotic (MCMA) Twin Pregnancies—Diagnosis, Prognosis and Management: Review of Literature

**DOI:** 10.3390/diagnostics13193109

**Published:** 2023-09-30

**Authors:** Pierpaolo Nicolì, Gianluca Raffaello Damiani, Maria Gaetani, Miriam Dellino, Amerigo Vitagliano, Antonio Malvasi, Gerardo Cazzato, Eliano Cascardi, Andrea Marzullo, Raffaello Alfonso, Ettore Cicinelli, Antonella Vimercati

**Affiliations:** 1 Department of Interdisciplinary Medicine (DIM), Unit of Obstetrics and Gynecology, University of Bari “Aldo Moro”, Policlinico of Bari, Piazza Giulio Cesare 11, 70124 Bari, Italy; 2 Private Practice Gynecologist, 70124 Bari, Italy; 3 Department of Precision and Regenerative Medicine and Ionian Area, University of Bari “Aldo Moro”, Policlinico of Bari, Piazza Giulio Cesare 11, 70124 Bari, Italy; 4 Department of Medical Sciences, University of Turin, 10124 Turin, Italy; 5 Pathology Unit, FPO-IRCCS Candiolo Cancer Institute, 10060 Candiolo, Italy

**Keywords:** monochorionic twin pregnancy, trap sequence, 3D ultrasound, transvaginal ultrasound, early diagnosis

## Abstract

The Twin Reversed Arterial Perfusion (TRAP) Sequence is an extremely rare complication of monochorionic twin pregnancies, with one severely malformed twin (the “acardiac”) lacking autonomous placental blood supply and being perfused by the co-twin (the “pump”), through arterio-arterial (and sometimes also veno-venous) vascular anastomoses located on the placental surface. The prognosis is poor: mortality is 100% in the acardiac twin because of its severe malformations and about 50–55% in the pump twin, mainly due to heart failure and prematurity. So, the goal of perinatal management of the TRAP twin pregnancy is to deliver a healthy and near-term pump twin without heart failure or fetal hydrops. Intuitively, the earlier the diagnosis, the better the outcome. Herein, we report two cases of monochorionic monoamniotic (MCMA) twin pregnancies complicated by the TRAP Sequence, which are of interest since the objective of early diagnosis was achieved by means of transvaginal and 3D ultrasound, two techniques which revealed themselves as being useful to this purpose but are underused in the literature. The second aim of this study is to provide an overview of literature data about the diagnosis, prognosis establishment, and management of this rare condition, which are still debated and unclear due to negligible poor-quality evidence.

## 1. Introduction

The Twin Reversed Arterial Perfusion (TRAP) Sequence, also known as *Chorioangiopagus parasiticus*, is an extremely rare complication of monochorionic twin pregnancies and the most severe form of Twin-to-Twin Transfusion Syndrome (TTTS) [1].

While van Gemert et al. [2] estimated that this condition complicates about 2.6% of monochorionic gestations and 1/9500–1/11,000 of all pregnancies, Quaas [3] suggested that the incidence of TRAP Sequence could be higher due to the increasing use of assisted reproductive technology and the early detection by first-trimester scan.

Although several theories have been postulated [4,5], the pathogenesis is still essentially unknown.

In this severe condition, one fetus, lacking autonomous placental blood supply, is perfused by the twin through arterio-arterial (and sometimes also veno-venous) vascular anastomoses located on the placental surface [6,7]. The donor twin is usually called “pump”, while the recipient is referred to as “acardiac”, since its heart is usually completely absent or, at most, a rudimental dysfunctional cardiac sketch is present. The pump twin has a normal circulatory pattern, but a part of its cardiac output is “stolen” by the acardiac twin, through the vascular anastomoses. It is a *reversed arterial perfusion* as the reflux blood from the circulation of the pump twin, so poor in oxygen, reaches the acardiac twin by pressure gradient through the umbilical artery (or arteries) and moves away through the umbilical vein, which is in the opposite direction to what usually happens. This circulation has negative consequences for both twins. The thin oxygen-poor blood flow stolen by the acardiac twin usually prevents its upper body formation, with the development of only lower extremity sketches. Specifically, the acardiac twin can assume four different anatomical forms [8]:

1. The most common subtype is the *Acardius acephalus*, in which the pelvis and lower limbs are well developed, the head and thoracic organs are missing, and poorly developed upper extremities may be present or absent.
2. The *Acardius anceps* is the most developed subtype, since both the upper and lower extremities are present and typically well-formed, and head and face are present too, although poorly formed.
3. The *Acardius amorphus* is the least developed subtype, a shapeless mass without differentiable organs, extremities, or anatomic structures.
4. The *Acardius acormus* is an extremely rare subtype; here, the head is the only recognizable organ.

On the other hand, the pump twin may develop polyhydramnios, cardiac failure and hydrops, up to death [9], due to its expanded cardiac demand. So, the fate of one twin appears to be inextricably bounded to that of the other one. Although there may be a certain balance between the parts at the beginning of pregnancy, the “bad” twin (the acardiac) could eventually take over the “good” one (the pump), leading to the death of the latter. This inevitably reminded us of the plot of the famous novel “The Strange Case of Dr Jekyll and Mr Hyde”.

Herein we report two cases of monochorionic monoamniotic (MCMA) twin pregnancies complicated by the TRAP Sequence which, in compliance with the patients’ preference, we managed with a conservative-only approach, even when early diagnosis was achieved by means of transvaginal and 3D ultrasound. Both our cases ended up as Stevenson’s literary bestseller: with the death of both protagonists. The second aim of this study is to provide an overview of literature data about the diagnosis, prognosis establishment, and management of this rare condition, which are still debated and unclear due to negligible poor-quality evidence.

## 2. Results

### 2.1. Case Report One

At 22 weeks of gestation, a 30-year-old female of Caucasian ethnicity with gravida 1 parade 0 abortion 0 (G1P0Ab0) was referred to our Obstetrics Department for the detection of a suspicious intramniotic amorphous mass during a routine second-trimester fetal anatomy scan. The patient, without a family history of twins, had conceived spontaneously. Anamnesis was silent for drug use, alcohol drinking, smoking, or pregnancy infection and she was healthy and with a normal BMI. During first-trimester ultrasound screening performed elsewhere, the acardiac fetus was not noted and the other one appeared to be normal. Then, the examiner who performed the second-trimester ultrasound anatomy screening detected the presence of a suspicious amorphous mass beside the apparently normal fetus, thus referring the patient to our secondary hospital.

**Figure 1:**
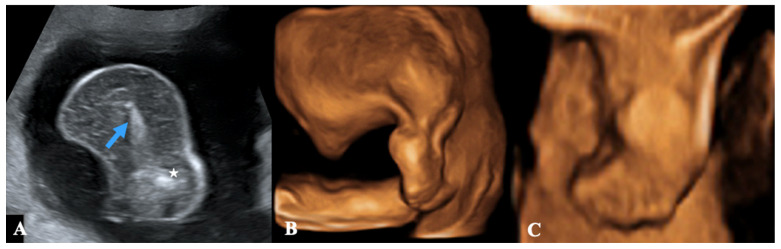
A transabdominal ultrasound in a 22-week pregnant patient. (**A**) A 2D ultrasonography (longitudinal view) showing an amorph mass with a lower limb consisting of femur (blue arrow pointing to part of the femur) and sketches of tibia and fibula (star). (**B**) A 3D ultrasonography (lateral view) confirming the presence of thigh, leg, and foot. (**C**) A 3D picture (frontal view) showing in detail the foot with five toes.

Our abdominal echography, performed by means of a GE Healthcare Voluson E10 ultrasound machine, revealed a monochorionic twin pregnancy with one acardiac fetus and one pump fetus. During the scan, we could not find a definite membrane separating the two twins; hence sonographically, we thought this twin pregnancy fell under the monochorionic monoamniotic twin pregnancy. The pump twin had a borderline hydrocephalus and a right aortic arch, but there were no signs of high-output cardiac failure (tricuspid regurgitation, cardiomegaly, pericardial and pleural effusion, ascites). As per the Williams package, the weight of the pump twin was 481 g (BPF = 5.29 cm; HC = 20.01 cm; AC = 17.37 cm; LF = 3.76 cm), consistent with menstrual age. The acardiac twin at the 2D abdominal ultrasound appeared to be an amorphous mass measuring 67 mm (maximum length), without the heart and with a lower limb consisting of femur and sketches of tibia and fibula (Figure 1A). It was also studied with a 3D abdominal ultrasound, which clearly confirmed the presence of a thigh, a leg (Figure 1B), and a five-toe foot (Figure 1C). The additional use of color Doppler sonography showed a reversal of blood flow in its umbilical artery, thus definitely confirming the diagnosis of TRAP Sequence with an *Acardius acephalus* twin (even if the pelvis and lower limbs were not completely developed). Lastly, ultrasound also revealed the presence of polyhydramnios with a 13 cm maximum vertical pocket of amniotic fluid.

**Figure 2:**
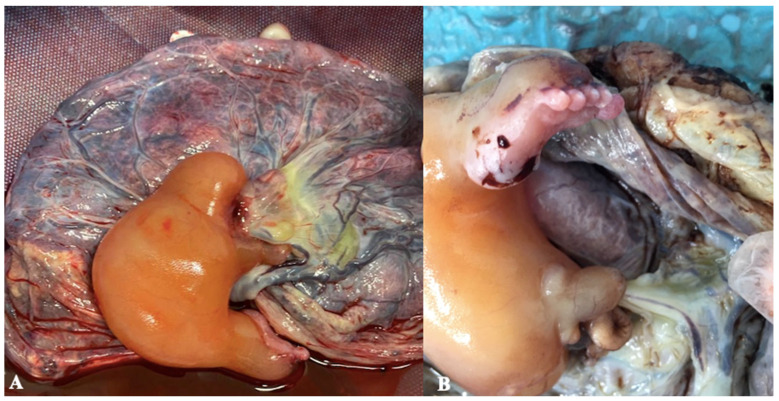
Histological specimens. (**A**) A macroscopic view of the acardiac twin and the placenta at birth. (**B**) The acardiac twin had only a lower limb (with femur and sketches of tibia and fibula) ending with a formed five-toe foot, and sketches of testicles and an oedematous penis.

Parents were extensively counselled about this rare condition, the possible prognosis, and all the different management alternatives (which we will discuss later in this article). The couple eventually refused invasive investigations and opted for elective termination of the pregnancy (TOP), which was performed uneventfully by prostaglandin induction. Fetal autopsy showed that the pump twin had no abnormal anatomy except a right aortic arch. Instead, the acardiac monster lacked cephalic extreme, main organs, and upper limbs (Figure 2A). It only had a lower limb (with femur and sketches of tibia and fibula) ending with a formed foot, and sketches of testicles and an oedematous penis (Figure 2B). It was directly connected to the placenta by an umbilical cord formed by a hypoplastic artery and a normal-sized vein. Examination of the placenta showed the presence on its surface of artery-to-artery and vein-to-vein anastomoses between donor and receiver, thus confirming the prenatal diagnosis of TRAP Sequence. The occurrence of anastomoses was based on morphological findings of large, ramified vessels surrounding the insertion of the umbilical cord and on histological features of the vessels demonstrating their arterial or venous nature. The membranes were confirmed to be monoamniotic–monochorionic.

### 2.2. Case Report Two

The second case we report concerns the prenatal diagnosis of a TRAP Sequence by means of transvaginal color Doppler imaging in early gestational age.

A 35-year-old female of South Asian ethnicity with gravida 1 parade 0 abortion 0 (G1P0Ab0) came to our hospital to undergo first-trimester ultrasound screening at 12 weeks of gestation. Pregnancy was spontaneous. The patient’s medical history was silent, and she had a family history of twin pregnancy.

**Figure 3:**
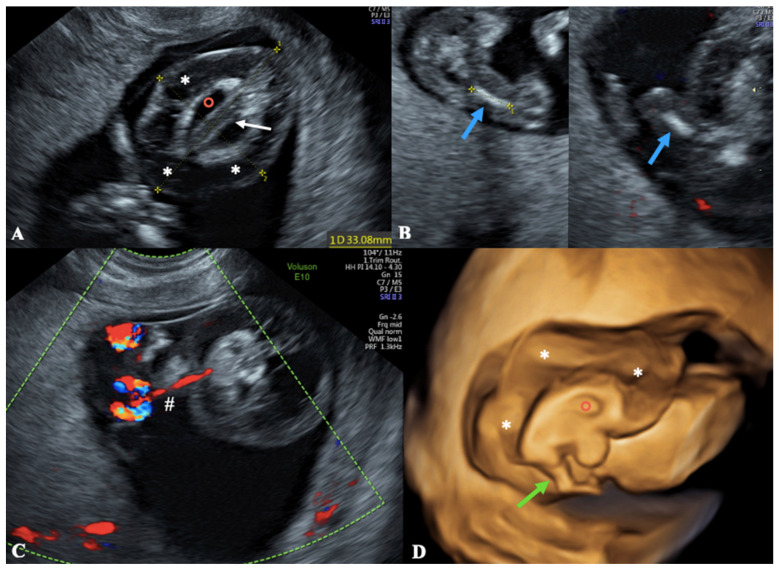
A transvaginal ultrasound in a 12-week pregnant patient. (**A**) The monster twin appeared as a hypo-hetero echoic mass with a rudimental vertebral column (white arrow) behind an anechoic cyst (red circle), and marked generalized edema of soft tissues (*). (**B**) An axial view of the monster twin showing femoral bones (blue arrows). (**C**) An Axial view of the monster twin highlighting retrograde umbilical perfusion (#) and absence of the heartbeat. (**D**) A sagittal view of the acardiac with 3D ultrasound technique showing anatomic details more clearly than the 2D standard way (the green arrow points to the sketch of a lower limb; * indicates the marked generalized edema of soft tissues).

Routine transabdominal ultrasound examination, performed by means of a GE Healthcare Voluson E10 ultrasound machine, revealed the presence of a morphologically normal fetus with crown-rump length (CRL) of 52 mm (sonographic age was consistent with menstrual age), fetal heart rate (FHR) of 145 bpm, and nuchal translucency of 1.1 mm. The placenta was posterior, the funiculus was formed by three vessels and the amniotic fluid volume appeared to be normal. Next to the fetus, the sonographer noticed the presence of a suspicious amorphous mass measuring about 33 × 25 mm which did not appear to be in continuity with the fetus but moved in a consensual manner to it. Therefore, the examiner performed a transvaginal ultrasound, and it became clear that the mass was a severely malformed fetus. Head, heart, main organs, and upper limbs were absent. The only recognizable anatomical structures were a rudimental vertebral column behind an anechoic cyst (Figure 3A) and the sketches of the lower limbs (with femurs and feet) (Figure 3B). The monster fetus also had marked generalized subcutaneous edema (Figure 3A). In this case too, the additional use of color Doppler sonography on the malformed fetus showed a reversal of blood flow in its umbilical artery, allowing us to diagnose a monochorionic twin pregnancy complicated by TRAP Sequence (Figure 3C) with an *Acardius acephalus* twin. The application of 3D ultrasound clearly showed the lower limb, the central anechoic cyst, and other anatomic details, thus confirming and better scanning the 2D ultrasound findings (Figure 3D). The absence of a membrane separating the two twins enabled us to deduce that the pregnancy was monochorionic-monoamniotic.

**Figure 4:**
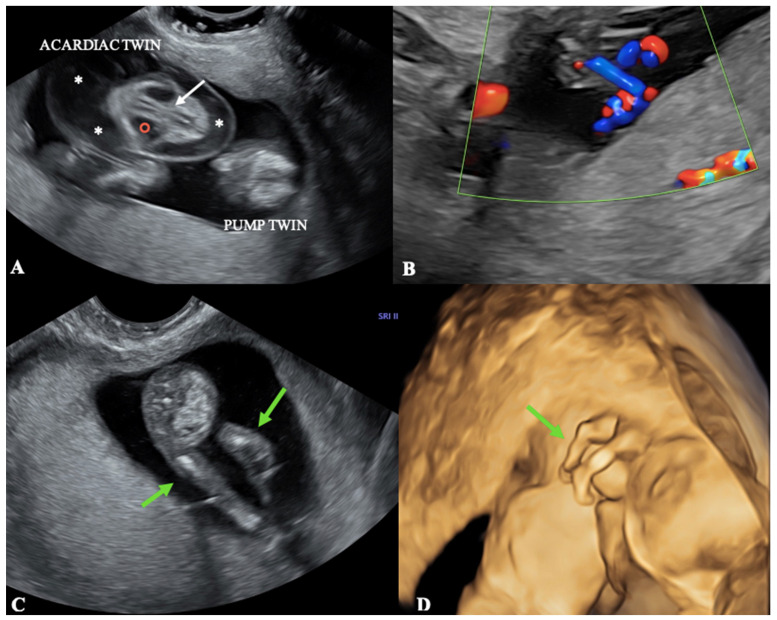
A transabdominal ultrasound in a 14-week pregnant patient. (**A**) In the 2D grayscale image, the monster twin appeared bigger when compared to the previous ultrasound, so the rudimental vertebral column (white arrow), the anechoic cyst (red circle), and the edema of soft tissues (*) were more evident. (**B**) The color Doppler study showed a marked perfusion into the umbilical cord of the acardiac fetus. (**C**) A 2D image showing the lower limbs of the acardiac twin (green arrows). (**D**) A 3D picture showing the lower limbs of the acardiac (green arrow) over the twin pump’s back.

The couple was scrupulously informed about this rare condition, the possible risks for both twins, and all the different management alternatives. They refused any invasive investigation and decided to do only observation first. We repeated ultrasound after two weeks, noting that the pump fetus had grown regularly (CRL = 7.7 cm) and, with color Doppler, that the perfusion into the umbilical cord of the acardiac fetus had not declined (Figure 4C), thus justifying that the monster twin had increased in size (about 55 × 37 mm) (Figure 4A). We recommended the patient underwent an invasive investigation, but she refused once again.

Unfortunately, the patient spontaneously miscarried two weeks later, before the subsequent check. The histological examination confirmed the MCMA pregnancy complicated by TRAP Sequence, based on the placental specimen. Due to technical reasons, the macroscopic pathologic images of the TRAP Sequence twins were unobtainable.

## 3. Discussion

The Twin Reversed Arterial Perfusion (TRAP) Sequence is an extremely rare complication of monochorionic twin pregnancies. Besides reporting two interesting additional cases, this study aims to contribute to the TRAP literature with a much needed summary of the existing evidence regarding three fundamental aspects of this rare condition, that is, diagnosis, prognosis establishment, and management. To the best of our knowledge, this is the first study of this kind, thus making it a potential key resource for all obstetricians, in particular those who will come across this pregnancy pathology for the first time.

### 3.1. Diagnosis

To be able to make a TRAP diagnosis firstly means to be able to differentiate an acardiac twin from a vanishing or severely malformed co-twin [10]. It requires adequate knowledge of this rare condition, encompassing both pathophysiological and anatomopathological aspects [11], but, most of all, remarkable sonographic skills [12].

To date, Color Doppler and pulse wave Doppler velocimetry studies represent the best methods with which to confirm the pathognomonic reversed blood flow into the acardiac twin through its umbilical artery [13,14,15,16]. Otherwise, the key findings of a monster fetus are multiple deep malformations, the lack of a heart, and massive edema of the soft tissues. Conversely, the pump twin may show growth restriction, flowmeter alterations in the umbilical artery and venous duct, signs of heart failure due to hypervolemic circulation (increased cardiac output, tricuspid regurgitation, cardiomegaly, pericardial and pleural effusion), polyhydramnios (due to increased perfusion of the fetal kidneys and, therefore, urine production), ascites, subcutaneous edema, and hydrops [9].

To the best of our knowledge, the diagnosis of acardia is almost always made in the second [17,18,19,20,21,22,23] or third trimester [24,25,26] of gestation by means of transabdominal ultrasound. In some cases, the existence of the TRAP Sequence could even remain unknown until delivery [27,28], when the childbirth is preceded or followed by ambiguous amorph mass expulsion. However, some authors [6,29,30,31,32,33] have reported TRAP diagnoses in early gestational age (11–12 weeks of gestation) by using the transvaginal method, as we did in Case Report Two. Although more difficult to realize, early diagnosis should be the goal, since on the one hand it gives the couples who receive a TRAP Sequence diagnosis and opt for elective TOP the chance to live an experience which is less traumatic than tardive termination or abortion would be; on the other hand, it increases the chance of a positive obstetrical outcome for the pump twin when pregnancy goes forward by applying early intervention, if appropriate.

Guimaraes and colleagues [34] used magnetic resonance imaging (MRI) to confirm the diagnosis early and establish the precise extent of fetal malformations in the TRAP Sequence.

The cases we reported are of interest since the diagnoses were achieved by means of 3D ultrasound too, which is a faster and a less expensive method than MRI, and does not require the help of a radiographer [35,36,37]. Thanks to 3D ultrasound, we observed the malformations with better definition and confirmed our original suspicions. The same advantages were also noticed by Bonilla-Musoles and coworkers [38]. Nevertheless, our review of the literature revealed that 3D ultrasound is underused to diagnose the TRAP Sequence.

### 3.2. Prognosis

The prognosis of monochorionic pregnancies complicated by the TRAP Sequence is poor: mortality is 100% in the acardiac twin [39,40] because of its severe malformations, and about 50–55% in the pump twin [9,39,40,41,42,43] mainly due to heart failure and prematurity, as reported by Sullivan and colleagues [44]. Unfortunately, there are no accepted criteria with which to predict adverse pregnancy outcomes of pump twins. We now summarize the most widely used criteria.

#### 3.2.1. Twins’ Weight Ratio

Some studies have shown that the acardiac twin size is correlated with the pump twin outcomes.

Jelin et al. [45] examined 18 acardiac twins (from many MCMA TRAP pregnancies) whose body weights were no more than 50% of those of the pump twins, finding that only one pump twin died in utero.

From the investigation of 13 cases of TRAP conducted in the Children’s Hospital of Philadelphia [46], it emerged that, when the body weights of the acardiac twins were no more than 40% of those of the pump twins, edema and heart failure were absent in the pump twins.

Also, Sepulveda and colleagues [47] believed that the twins’ weight ratio was an essential prognostic factor for the TRAP outcome.

Moore and coworkers [9] suggested that the prognosis of the pump twin is directly related to the size of the acardiac one and they came up with a second-order regression equation to deduce the weight (in gm) of the monster twin:

Weight = 1.2L^2^ − 1.7L, (1)

where L is the maximum length of the acardiac twin. According to Moore, if the ratio between the weight of the acardiac twin and that of the pump twin (APTW) is greater than 70:100 (0.7), the incidences of preterm delivery, polyhydramnios, and heart failure for the pump twin are 90%, 40%, and 30% respectively; when APTW is lower than 0.7, the incidences are 75%, 30%, and 10% [9]. In the literature, Moore’s weight comparison between the monster and the normal twin has widely been used with sound confirmations of its goodness for predicting the outcome of TRAP pregnancies [18,19,20,22,24]. If we wanted to apply Moore’s concept in our Case Report One, the estimated weight of our anomalous twin, based on a maximum length of 6.7 cm, was approximately 42.5 g. As per the William package, pump twin measured 480 g (Figure 3A), yielding a 0.09 ratio. Although the pump twin was apparently fine (apart from the right aortic arch and a borderline hydrocephalus) and, according to Moore’s equation, prognosis would have been good, the couple refused a conservative approach and opted for elective TOP.

Regarding early pregnancy, some studies reported that ultrasound indicators such as pump twin’s CRL and acardiac twin’s upper pole-rump length (URL) carefully indicated the pump twin outcomes. The

(CRL − URL)/CRL(2)

and

URL/CRL(3)

ratios were shown to be significantly associated with adverse pregnancy outcomes of the pump twins [32,48]. Tang et al. [49] recently conducted an observational prospective study on 21 TRAP pregnancies diagnosed early with the aim of confirming those results. They found that a (CRL − URL)/CRL ratio > 0.43 and/or a URL/CRL ratio < 0.57 identified the pump twin survivors, and their multiple regression analysis showed that the two ratios were significantly associated with the pump twin’s survival after controlling for the other variables. If we consider Tang’s cutoff, the application of this concept to our Case Report Two at 12 pregnancy weeks ((CRL − URL)/CRL = 0.37; URL/CRL = 0.63) and at 14 pregnancy weeks ((CRL − URL)/CRL = 0.29; URL/CRL = 0.71) further confirms this correlation and is consistent with the prognosis we observed in this case (spontaneous miscarriage).

#### 3.2.2. Twins’ Vascular Disjunction

A spontaneous interruption of the vascular connection between the twins has been shown to occur [25,28,33,50], with the acardiac twin remaining smaller than the other one and the pump twin outcome being excellent [46].

Chen et al. [51] reported seven cases of spontaneously blocked TRAP, with three cases of intrauterine demise and one case of polyhydramnios-induced premature birth.

Tang et al. [49] identified five TRAP cases with naturally blocked blood flow, of which four cases survived. In one case, the pump twin developed polyhydramnios within one week after the blood flow block.

As far as we are concerned, we did not document the twins’ vascular connection weakening in our Case Report Two, and at the second sonographic check, the reversed arterial perfusion to the acardiac was still present and well evident (Figure 4B). This was consistent with the evidence that the monster had grown as compared to the previous visit and confirms that the persistence of connection between the twins worsens pregnancy prognosis. Indeed, about two weeks later our patient spontaneously miscarried.

#### 3.2.3. Power Doppler Velocimetry Data

Few studies [51,52] have indicated that the resistance index of the umbilical artery and the pulsatility index of the middle cerebral artery of the donor twin may be indicators with which to assess the TRAP prognosis at a gestational age greater than 20 weeks.

### 3.3. Management

To provide potential prognosis predictors is essential because it improves the physician’s likelihood of choosing the best management for *that* TRAP pregnancy, which still remains the subject of scientific debates. The goal of perinatal management of the TRAP twin pregnancy is to deliver a healthy and near-term pump twin without heart failure or fetal hydrops. We present an overview of the possible management approaches.

#### 3.3.1. Conservative Approach

The first option the couple has is the observational-only approach, with possible study of the fetal karyotype (the pump twin could be affected by diseases that cannot be sonographically detected [53,54,55]), serial ultrasound checks, and amnioreduction in the case of polyhydramnios. Furthermore, since previous studies have shown that malformations affecting the heart are the most common ones in multiple gestations (3.8% is the estimated incidence [56]), with a much higher rate in monochorionic pregnancies, a fetal echocardiography is always recommended [57].

Sullivan and colleagues [44] believed that the best initial treatment is expectant management since the cessation of blood flow between fetuses may occur spontaneously. However, 40% of cases reported in their study were associated with a very small acardiac twin with a postpartum APTW ratio of less than 3%.

One study [58] conducted in Australia showed that the survival rate of the pump twin was more than 80% after the expectant management of the TRAP.

Also, Jelin et al. [45] and Mann et al. [46] preferred to perform expectant management.

#### 3.3.2. Interventional Approach

An alternative to expectant management is the prophylactic interventional approach, with the aim of interrupting blood flow from the pump twin to the acardiac one. Several minimally invasive procedures have been progressively introduced [59] and, to date, the most common technique reported in the literature for the treatment of the TRAP Sequence is radiofrequency ablation (RFA), under ultrasound or fetoscopic guidance [59].

However, to date, there is no consensus about timing for intervention in this pathology. Since TRAPs are almost always diagnosed in the second trimester of pregnancy, most of them underwent treatment at 16 weeks or more. Nowadays, early diagnosis is more and more frequent, so it is fundamental to understand whether immediate intervention could and should be taken after diagnosis. Several studies seem to suggest that interstitial laser ablation (ILA) is a technically feasible treatment for the TRAP in the first or early second trimester (before 16w) [48,60,61]. On the other hand, Lewi and colleagues [32] argue that (i) the total number of patients in these studies is small, (ii) there is a significant bias risk, and (iii) the fetal loss rate with operative management exceeds the fetal loss rate with observational management. We know that in 2016, a multicenter randomized controlled trial started (https://clinicaltrials.gov/ct2/show/NCT02621645, accessed on 15 September 2023), aiming to clarify whether early intervention (12–14 weeks, study group) leads to better pregnancy outcomes as compared to later intervention (16–19 weeks, control group). The results of this study should be available to the public within the next months.

Regardless of the gestational age, unexpected death of the pump twin, iatrogenic premature rupture of the membranes, and preterm birth are some of the complications that can occur after mini surgery [59]. Furthermore, like all monochorionic-monoamniotic pregnancies, also those complicated by TRAP are at greater risk of intrauterine fetal demise due to cord entanglement, still possible even after a successful cord occlusion/ablation in TRAP cases [62,63]. Although data supporting this approach are not yet robust, to prevent this sneaky complication, numerous groups reported performing cord transection of the reduced twin following cord occlusion/ablation [64,65]. It is typically performed by coagulating the cord in at least two locations and then transecting between them, by using forceps or laser.

#### 3.3.3. Mixed Approach

Finally, some authors suggested that a conservative approach should be the first choice, with the possibility of shifting to intervention to stop the reversed perfusion in case signs of fetal impairment develop, for example in the case of polyhydramnios appearance, in case the abdominal circumference of the acardiac twin becomes equal to or greater than that of the pump twin, or in the case of abnormal blood flow into the pump twin [45,63,66].

All of Tang’s [49] TRAP patients first underwent the observational-only approach, until six cases (among the 21 patients) were subjected to the mini-invasive bipolar electrocoagulation in the umbilical cords of the acardiac twins at 24–26 weeks of gestation (when edema, heart failure, and polyhydramnios in the pump twin occurred or the body weight of the acardiac twin became greater than 50% of that of the pump twin). Overall, 66.7% (*n* = 14) of the pump twins were born healthy.

To assist with the decision of *when* to shift to treatment, Wong and Sepulveda [15] suggested a new classification based on the size and growth of the acardiac and on the cardiovascular condition of the pump twin. When the acardiac twin is small and signs of cardiovascular impairment in the pump twin are absent, they recommend serial ultrasound checks to detect any worsening. In cases with a large acardiac twin or rapid growth of the acardiac mass, they advise the shift to an interventional approach.

Malone and D’Alton [67] suggested expectant management with weekly sonographic checks in the case of (i) APTW < 0.70, (ii) a lack of echocardiographic heart failure in the pump twin, and (iii) well represented amniotic fluid volume.

Clifton O. Brock. et al. [59], in their recent review, recommended a weekly evaluation of the following parameters: (1) the growth trend of both twins; (2) Doppler velocimetry in the umbilical artery, middle cerebral artery, and venous duct in the pump twin; (3) detection of blood flow and Doppler velocimetry in the umbilical artery of the monster twin; (4) the cardiac output of the pump twin; and (5) the maximal vertical pocket of amniotic fluid (with particular regard to the sac of the pump twin in cases of diamniotic pregnancy). If there are no complicating factors and the pump twin does not have signs of impairment, they consider a delivery after 34 weeks reasonable [59].

However, the couples who decide to take a conservative approach should know that there are no clear and unequivocal data yet indicating the right frequency of clinical checks and the right parameters to analyze during each one.

The choice between the two main approaches is not simple, since on the one hand there is the risk of the possible progressive deterioration of the cardiac function of the pump twin, as well as the risk of preterm birth in the case of polyhydramnios and the risk of intrauterine fetal demise; on the other hand, the fetal complications from mini-surgery (unexpected death of the pump twin, iatrogenic premature rupture of the membranes, preterm birth) and all maternal risks (intrauterine infection, hemorrhage, sepsis, burn damage, etc.) must be considered.

Both our cases were managed in compliance with the patients’ preferences, though these latter were not consistent with the most recent scientific data. The first patient was extensively informed that the prognosis would have been good and, anyway, she would have been eligible for the mini-invasive surgery; despite explaining that her choice was not aligned with the recent literature evidence, she was still determined to achieve an elective TOP, thus we respected her desire. Regardless of the estimated prognosis, the second patient was unmovable in her decision of a conservative-only approach, based on ethical reasons.

**Figure 5:**
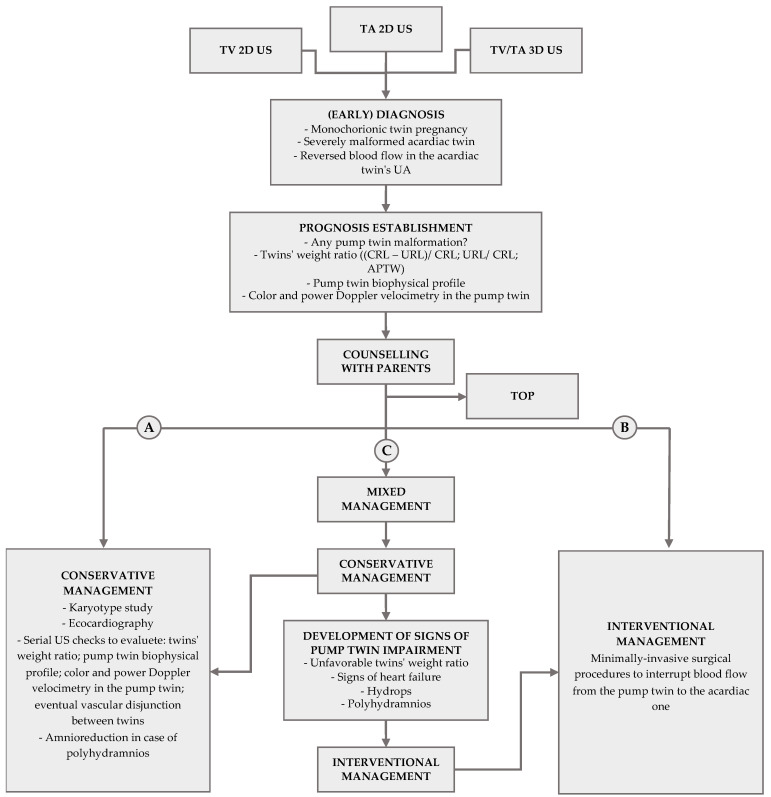
The picture displays a flowchart summing up how a monochorionic twin pregnancy complicated by TRAP can be managed. TA = transabdominal; TV = transvaginal; US = ultrasound; UA = umbilical artery; TOP = termination of the pregnancy.

Table 1 provides an overview of the largest groups of TRAP pregnancies reported in the literature, together with relative management and outcomes. The subsequent Figure 5 displays a flowchart summing up how a monochorionic twin pregnancy complicated by TRAP can be managed.

## 4. Conclusions

The Twin Reversed Arterial Perfusion (TRAP) Sequence is a rare, severe condition, which should always be suspected in monochorionic twin pregnancies if one of the twins is acardiac.

The goals should be early diagnosis and good clinical counselling in order to ensure, in compliance with the patients’ preference, an earlier and more adequate treatment to reduce the otherwise extremely high risk of intrauterine fetal demise for the pump twin, and to improve the prognosis.

Early diagnosis can be more easily achieved in second-level centers by operators with remarkable sonographic skills and by means of the transvaginal technique and 3D ultrasound. Although not indispensable to achieve a TRAP diagnosis, these latter may be extremely useful and complementary to the traditional transabdominal ultrasound.

Parents should be immediately informed about the estimated prognosis of the donor fetus and all possible management modalities in order to allow them to choose the fate of their pregnancy freely and consciously.

Due to negligible and poor-quality evidence, which prevents guidelines from being drawn up, future carefully designed randomized controlled trials and prospective studies comparing diagnostic modes, prognostic indicators, and management approaches are still needed.

## Figures and Tables

**Table 1:** The largest studies reported in the literature regarding TRAP management and relative outcomes.

First Author, Year	Study Type	No of Treated Cases	GA at Diagnosis (Weeks)	Management (GA at Int, Weeks)	Outcome
Moore et al., 1990 [9]	Retrospective	49	ND	Con	24 Good, 20 IUFD, 5 PD
Quintero et al., 1996 [68]	Prospective	10	ND	Int (mean 21, range 16–25)	5 Good, 4 IUFD, 1 PD
Sullivan et al., 2003 [44]	Prospective	10	Mean 20.3 (range 19–24)	Con	9 Good, 1 IUFD
Weisz, 2004 [66]	Retrospective	6	>14.0 (range 14–23)	Mixed (3 Int, ND)	5 Good, 1 IUFD
Quintero et al., 2006 [63]	Prospective	65	ND	14 Con	6 Good, 8 IUFD
				51 Int (mean ± SD, 21.2 ± 2.5)	33 Good, 18 IUFD
Livingston et al., 2007 [69]	Prospective	17	ND	Int (mean 21, range 17–24)	12 Good, 1 UFD, 4 ND
Lewi et al., 2010 [32]	Retrospective	24	Range 11.0–13.6	Mixed (10 Int, range 16–18)	11 Good, 12 IUFD, 1 TOP
Jelin et al., 2010 [45]	Retrospective	18	ND (only abstract available)	11 Con	10 Good, 1 IUFD
				7 Int (ND)	7 Good
Scheier et al., 2012 [70]	Retrospective	9	Range 12.0–20.3	Int (median 16.2, range 13.1–20.3)	7 Good, 2 IUFD
Pagani et al., 2013 [71]	Retrospective	23	Median 13.3 (IQR 12.1–19.4)	6 Con	6 IUFD
				17 Int (median 18.4, IQR 15.1–21.5)	14 Good, 3 IUFD
Lee et al., 2013 [72]	Retrospective	98	Mean ± SD, 17.9 ± 3.2	Int (mean ± SD, 20.2 ± 2.4)	78 Good, 20 IUFD
Berg et al., 2014 [60]	Retrospective	Group A, 23	Mean ± SD, 19.9 ± 6.3	16 Con	13 Good, 3 IUFD
				7 Int (mean ± SD, 23.0 ± 5.0)	6 Good, 1 IUFD
		Group B, 17	Mean ± SD, 16.4 ± 4.7	6 Con	5 Good, 1 IUFD
				11 Int (mean ± SD, 23.5 ± 2.6)	8 Good, 3 IUFD
Takano, 2015 [65]	Retrospective	10	ND	Int (median 21.3, range 16.7–27.3)	9 Good, 1 IUFD
Fisher et al., 2016 [58]	Retrospective	15	Mean ± SD, 18.5 ± 3.9	8 Con	8 Good
				7 Int (mean ± SD, 23.5 ± 2.6)	6 Good, 1 IUFD
Sugibayashi et al., 2016 [62]	Retrospective	40	Mean ± SD, 18.7 ± 3.2	Int (mean ± SD, 20.8 ± 2.2)	34 Good, 5 IUFD, 1 PD
Roethlisberger et al., 2017 [48]	Retrospective	12	Median 12.8 (IQR 12.1–13.3)	Int (Median 13.2, IQR 12.6–13.6)	7 Good, 5 IUFD
Tavares de Sousa et al., 2020 [61]	Retrospective	12	<14.0	Int (Median 13.5, IQR 13.4–14.0)	11 Good, 1 IUFD
Weber et al., 2021 [73]	Retrospective	15	<14.0	8 Con	8 IUFD
				7 Int (ND, only abstract available)	4 Good, 7 IUFD
Tang et al., 2022 [49]	Prospective	21	Mean ± SD, 13.1 ± 0.18	Mixed (6 Int, ND)	14 Good, 7 IUFD

GA = gestational age; Int = interventional; Con = conservative; IUFD = intrauterine fetal demise; PD = perinatal death; ND = No data; IQR = interquartile range.

## Data Availability

All data are reported in the text.
